# Mindfulness and acceptance-based training for elite adolescent athletes: a mixed-method exploratory study

**DOI:** 10.3389/fpsyg.2024.1401763

**Published:** 2024-05-27

**Authors:** Ning Su, Gangyan Si, Wei Liang, Danran Bu, Xiaobo Jiang

**Affiliations:** ^1^Physical Education School, Shenzhen University, Shenzhen, China; ^2^Sport Psychology Centre, Hong Kong Sports Institute Limited, Hong Kong SAR, China; ^3^School of Physical Education, Hubei University, Wuhan, China

**Keywords:** mindfulness and acceptance-based training, elite adolescent athletes, mixed-method, sport training performance, Chinese

## Abstract

**Objectives:**

This study aimed to examine the effectiveness of a specifically designed mindfulness-acceptance-insight-commitment (MAIC) training program on relevant psychological factors (i.e., mindfulness, acceptance, performance-related satisfaction) as well as sport training performance for elite adolescent athletes from Hong Kong. And it also aimed to explore the athletes’ real experiences (i.e., receptiveness and perceptions) of completing the MAIC program.

**Methods and design:**

The mixed-method was used in this study, including a randomized controlled trial (RCT) and a qualitative exploration. The RCT employed a 2 (groups) x 3 (data collection points) design involving 40 elite adolescent athletes from the Hong Kong Sports Institute (HKSI). These athletes were randomly assigned to either the MAIC training group (MT; *n* = 20, *Mage* = 15.65) or the control group (CG; *n* = 20, *Mage* = 15.85) to further test the effectiveness of the MAIC intervention on mindfulness, acceptance, performance-related satisfaction, and sport training performance. Subsequent to the RCT, the qualitative exploration was used to explore the athletes’ real experiences towards the MAIC program. In the qualitative exploration, all athletes who participated in the MAIC program were invited to participate in voluntary semi-structured interviews. Of these, 14 athletes chose to take part in the interviews. The RCT employed a 2×3 mixed-design ANOVA, while thematic analysis was applied to the qualitative exploration.

**Results:**

The results revealed that the MAIC training program significantly enhanced athletes’ mindfulness, acceptance, satisfaction with performance, and sport training performance. However, these effects diminished at the follow-up assessment compared to post-training. Notably, the acceptance level of MT athletes did not significantly differ from CG athletes at the follow-up assessment. Additionally, the qualitative analysis identified four key dimensions: (a) Attitude towards MAIC training, (b) Reflection on the MAIC learning process, (c) Outcomes of MAIC training, and (d) Recommendations for future MAIC training. Overall, the qualitative findings complemented and reinforced the quantitative results, offering deeper insights into athletes’ experiences and valuable suggestions for further enhancing the MAIC program.

**Conclusion:**

The findings suggested that the specifically designed MAIC training program in this study effectively enhanced sport training performance and various psychological factors among elite adolescent athletes from Hong Kong. Nevertheless, further investigations are still required to comprehensively evaluate and further develop the MAIC training program.

## Introduction

Elite sports is a domain replete with formidable challenges and intense competition. Athletes within this realm tirelessly endeavor to attain optimal performance states and consistently surpass high performance benchmarks. In pursuit of their goals, it is indisputable that each elite athlete confronts various stress and challenges throughout their sporting careers. Over the past four decades, traditional psychological skills training (PST), primarily influenced by cognitive-behavioral theories [e.g., Meichenbaum (1977) and Bandura (1997)], has been the prevailing approach in sport psychology aimed at aiding athletes in enhancing performance and effectively navigating these challenges.

The PST focuses on self-control of internal processes, such as thoughts, feelings, and bodily sensations (Harvey et al., 2002). The psychological interventions stemming from the PST have been utilized to develop athletes’ mental skills such as imagery, goal setting, arousal control, self-talk, precompetitive routines and so on. These control-based cognitive-behavioral interventions aim to optimize performance states by aiding self-regulation of internal processes such as confidence, attention, emotion, cognition, and physiological states (Hardy et al., 1996). Throughout the decades, PST has been the predominant method for enhancing sport performance. However, despite over 30 years of both theoretical development and practical application, research findings from various studies [e.g., Maynard et al. (1995), Thelwell and Greenlees (2003), Mamassis and Doganis (2004), Birrer and Morgan (2010), and Barker et al. (2020)] suggest that traditional PST approaches may effectively modify variables believed to influence athletic performance, but fail to produce significant improvements in actual athletic performance (Zaichkowsy and Baltzell, 2001; Gould et al., 2002). In other words, the efficacy of PST seems limited or questionable for pursuing the optimal athletic performance.

In light of traditional Psychological Skills Training (PST) limitations, mindfulness-based interventions have gained traction in sport psychology as promising alternatives (Gardner and Moore, 2007). Initial applications of mindfulness in sports, pioneered by Kabat-Zinn et al. (1985), showed collegiate rowers exceeding expectations and Olympic medalists crediting mindfulness for their success. Despite a hiatus, recent years have witnessed a resurgence in mindfulness-based training programs, with empirical studies demonstrating their effectiveness in enhancing performance (Birrer et al., 2012; Gardner and Moore, 2012; Baltzell and Akhtar, 2014; Baltzell et al., 2015). Unlike PST, mindfulness approaches emphasize cultivating nonjudgmental awareness and acceptance of internal experiences, aiming to enhance psychological flexibility (Gardner and Moore, 2004, 2007; Sappington and Longshore, 2015). In recent years, with the widespread adoption and success of mindfulness in Western psychology, mindfulness-based programs have begun to attract the attention of Chinese scholars (Si et al., 2016). One such program, the Mindfulness-Acceptance-Insight-Commitment Training (MAIC), has been tailored specifically for Chinese athletes by the authors, and the first manual was published in 2014 (Si et al., 2014). During the passed four decades, various mindfulness-based programs have been developed and utilized by Western sport psychologists, with notable examples such as the Mindfulness-Acceptance-Commitment (MAC; Gardner and Moore, 2007) and the Mindfulness Sport Performance Enhancement (MSPE; Kaufman et al., 2009). However, these programs are designed primarily for athletes from Western cultural backgrounds. To ensure better effectiveness, it is imperative to consider indigenous cultural factors when designing mindfulness-based training for athletes from diverse cultural backgrounds (Si et al., 2016).

The MAIC, building upon the established MAC protocol (Gardner and Moore, 2007), integrates the concept of “insight” derived from Chinese Zen Buddhism and incorporates Si′s acceptance-based adversity coping model (Si, 2006). Insight, in this context, refers to a direct, non-conceptual understanding attained through repetitive contemplation of meditation objects. Within Chinese cultural understanding, insight represents a newfound awareness or revelation in life, demonstrated by individuals achieving a deeper comprehension of life’s meaning and personal values. The essence of insight lies in individuals’ dedication to and engagement with current tasks, achieved through detachment, symbolizing liberation from mental fixation and allowing for a clearer understanding of one’s motivations and values (Si et al., 2016). Si (2006) emphasizes peak performance not as perfection but as effective coping with adversities through acceptance and learning. Grounded in Chinese Zen Buddhism, acceptance-based coping underscores athletes’ capacity to coexist with adversities (Si et al., 2010). The MAIC places emphasis on acceptance training over altering mental states, viewing adversity as inherent to competition (Si et al., 2016). As a structured mindfulness and acceptance-based training regimen designed to enhance athletes’ performance and overall well-being, the MAIC comprises seven core components: (a) Introduction and psychoeducation of the MAIC, (b) Mindfulness, (c) Decentering, (d) Acceptance, (e) Values and insight, (f) Commitment, and (g) Comprehensive review and consolidation (Si et al., 2014). Although the MAIC has demonstrated promising outcomes in elevating athletes’ mindfulness and acceptance levels and improving athletic performance in various studies [e.g., Bu and Si (2014) and Si et al. (2016)], these studies have primarily been confined to case studies. Further randomized controlled trials are imperative to fully establish the efficacy of the MAIC in enhancing athletes’ performance and overall well-being. Exploration of mindfulness-based training for Chinese athletes is still in its nascent stages (Huang and Su, 2017). Moreover, there is currently a dearth of published research on the application of mindfulness-based training among elite athletes in Hong Kong. Elite adolescent athletes in Hong Kong encounter challenges not only in their athletic pursuits but also in their academic endeavors (Cheung and Chiu, 2016). Hence, offering mindfulness-based training to augment their athletic performance and overall well-being, particularly through the culturally adapted MAIC program, is necessary and significant due to the growing body of evidence demonstrating its effectiveness in enhancing key psychological skills, promoting mental health, and improving performance outcomes in athletes. Research into the implementation of the MAIC with elite adolescent athletes in Hong Kong can provide further insights into its effectiveness. Furthermore, some scholars have proposed that mindfulness-based training should be tailored to be more accessible and user-friendly for diverse populations (Baltzell and Akhtar, 2014). Therefore, this study aims to investigate the efficacy of the MAIC program on pertinent psychological factors (i.e., mindfulness, acceptance, performance-related satisfaction) and sports training performance among elite adolescent athletes from Hong Kong, as well as to explore athletes’ real experiences (i.e., receptiveness and perceptions) of completing the MAIC program.

## Methods

### Participants

In this study, 40 athletes aged 14 to 19 (*M_age_* = 15.75, *SD_age_* = 1.31; females = 17, males = 23), who had not previously received any form of mindfulness-based training, and had a minimum of 2 years of training experience were recruited from the elite sport teams Wushu and Tenpin Bowling of the Hong Kong Sports Institute (HKSI). Among these participants, 20 athletes (*M_age_* = 15.65, *SD_age_* = 1.39; females = 9, males = 11) were randomly assigned to the Mindfulness Training (MT) group, while the remaining 20 athletes (*M_age_* = 15.85, *SD_age_* = 1.23; females = 8, males = 12) were assigned to the Control Group (CG). All participants completed the study and data collection procedures, and there were no dropouts during the course of the study.

Prior to recruitment, the research plan was thoroughly explained to the relevant head coaches, and informed consent was obtained from interested coaches, athletes, as well as parents or legal guardians before participation. Participation was voluntary, and athletes had the option to withdraw from the study at any time. Furthermore, confidentiality and anonymity of their data were assured and respected throughout the study.

### Design

A quantitative approach was employed to examine the effectiveness of the MAIC on athletes’ sport training performance and relevant psychological factors, namely mindfulness, acceptance, and performance-related satisfaction. A 2×3 mixed design was utilized, with experiment condition as the between-subjects factor (comprising 2 levels) and test point/time as the within-subjects factor (comprising 3 levels). Data collection took place for both groups at pre-, post-, and 2 months following the MAIC training, after which the collected data were analyzed.

A qualitative approach was employed to explore the athletes’ experiences with the MAIC training, focusing on their receptiveness to the program and their perceptions of its impact. Following the completion of the MAIC training, all athletes from the MT group were invited to participate in semi-structured one-on-one interviews. The interview contents were then subjected to qualitative analysis.

### Procedure

Firstly, the entire research plan was presented to the relevant head coaches. Following the receipt of informed consent from interested head coaches and athletes, 40 elite adolescent athletes were recruited and randomly assigned to one of two groups (MT and CG). 20 athletes in the MT group received and successfully completed the MAIC training within a two-month period. Throughout these 2 months, the remaining 20 athletes in the CG group did not receive any psychological interventions. However, for equitable treatment, the opportunity for MAIC training would be extended to the CG athletes after the entire study completed. Furthermore, the adolescent athletes were grouped to undergo sports training at different times, ensuring that MT and CG athletes participated in separate sessions. This organizational strategy was implemented to mitigate the potential for cross-group contamination.

Data collection was conducted at three points in time: before the start of the MAIC training, immediately following the completion of the training, and 2 months post-training [a time frame supported by previous studies, e.g., Si et al. (2016)]. This collection involved assessing athletes’ performance through coach ratings and self-assessments, as well as evaluating psychological factors such as mindfulness level, acceptance level, and performance-related satisfaction.

Following the conclusion of the MAIC training, immediately, all participating athletes in the MT group were invited to participate in semi-structured interviews to discuss their experiences with the program. Fourteen athletes from the MT group volunteered to participate in these interviews. Each interview took place in a serene and confidential environment, typically the first author’s office, with a duration of approximately 30–45 min. All interviews were conducted within a time frame of no more than 2 months after the conclusion of the MAIC training.

### Intervention

The MAIC program comprised seven sessions, completed within a span of 2 months. Across the initial 7 weeks, MAIC training sessions were held weekly, each lasting approximately 60 min. The program culminated in a comprehensive discussion during the final week. The details of sessions in the MAIC training program please see Table 1.

**Table 1:** Details of sessions in the MAIC training program.

Time	Theme of Session	Content
Week 1	Introduction and psycho-education	Introduction of the entire structure of MAIC; Introduction of the theoretical rationale and specific goals; Introduction of acceptance-based adversity coping; Story example; Practice of brief centering exercise; Q & A about practice, explanation; Homework
Week 2	Introducing and practicing mindfulness	Introduction of mindfulness (“as it is” & “here and now”); Story example; Practice of mindfulness (e.g., mindfulness breathing, mindfulness walking, mindfulness yoga, mindfulness eating, etc.); Q & A about practice, explanation; Homework
Week 3	Introducing and practicing decentering	Introduction of decentering; Ruminated self-orientation to decentered task-orientation; Story example; Mindfulness exercises such as forgetting-self behavior exercise; Q & A about exercise, explanation; Homework
Week 4	Introducing and practicing acceptance	Introduction of acceptance; Using acceptance-based adversity coping to facilitate understanding acceptance and avoidance of experiences; Acceptance and nonjudgement of adversity or distractions; Story example; Coexistence exercises; Q & A about exercise, explanation; Homework
Week 5	Introducing value and insight	Introduction of value and insight; Understanding the relationship among value, insight, mindfulness, and acceptance; Story example; Instruction of insight to find out value; Q & A about exercise, explanation; Homework
Week 6	Introducing commitment	Introduction of commitment; Commitment for facing adversity and distractions; Linking commitment to value, insight, mindfulness, and acceptance; Story example; Q & A about exercise, explanation; Homework
Week 7	Comprehensive review and consolidation	Summary and overall understanding of the MAIC; Practice of key exercises and link them up; Explanation of the requirement for continuous commitment
Week 8	General discussion	General and open discussion about the entire MAIC

### Measures

***Athlete Mindfulness Questionnaire*** (AMQ; Zhang et al., 2017) is a 16-item questionnaire measuring athletes’ mindfulness levels during both training and competition from three dimensions: (a) present-moment attention, (b) awareness and (c) acceptance. Items are rated on a five-point Likert scale ranging from 1 (never true) to 5 (always true). Subscale scores for each dimension can be calculated, with a higher composite score across all dimensions indicating a greater level of mindfulness. The internal consistent reliabilities of the present-moment attention (*ρ* = 0.75), awareness (*ρ* = 0.76), and acceptance (*ρ* = 0.64).

***Chinese Version of Acceptance and Action Questionnaire-II*** (CV-AAQ II; Zhang et al., 2014) is a seven-item single-dimensional self-report questionnaire to measure one’s levels of experiential avoidance and psychological inflexibility (the opposite of psychological flexibility). Items are rated on a seven-point Likert scale ranging from 1 (never true) to 7 (always true), with low scores indicating a low level of experience avoidance and psychological inflexibility (i.e., high level of acceptance). The Chinese version of the AAQ-II demonstrated a high level of internal consistency and reliability in two samples of Chinese college students (composite reliability: *ρ* = 0.89 and *ρ* = 0.88) and a sample of elite Chinese athletes (*ρ* = 0.85).

***Training and Competition Satisfaction Scale*** (TCSS; Zhang and Liang, 2002), a 6-item scale, was employed to assess athletes’ satisfaction levels regarding their training or competition. Each item is rated on a 7-point Likert scale, ranging from 1 (strongly disagree) to 7 (strongly agree). The internal consistency of the TCSS is *α* = 0.75. Higher scores reflect greater levels of satisfaction with training or competition experiences.

***Coach-Rating Performance Scale*** and ***Athlete Self-Rating Performance Scale*** (CRPS and ARPS) were developed based on performance criteria specific to various sports and relevant studies (e.g., Liu et al., 2016; Si et al., 2016), following discussions with four relevant coaches involved in the research. Both CRPS and ARPS were designed to evaluate athletes’ performance across three facets: (a) training commitment, (b) movement qualities, and (c) movement stability. Each facet encompassed several sub-aspects, such as training attendance and duration in facet (a), movement accuracy and difficulty in facet (b), and error frequency and completion percentage of required actions in facet (c). Coaches rated the CRPS on a 10-point Likert scale ranging from 1 (worst) to 10 (best), while athletes rated the ARPS on the same scale. Scores for both CRPS and ARPS were obtained by summing the scores across the three facets.

### Interview guideline

**Figure 1:**
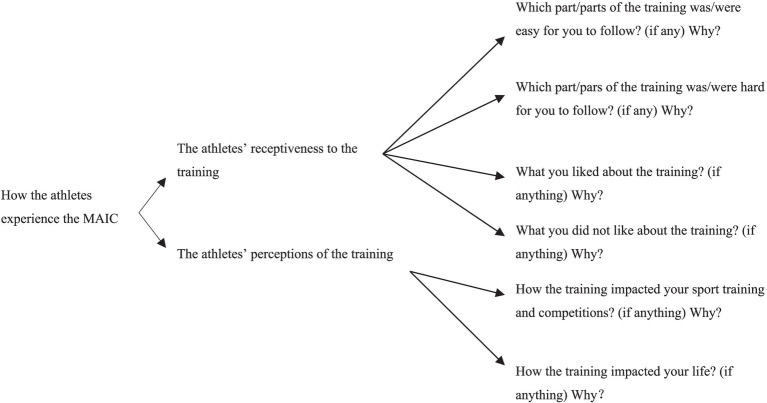
The interview map.

Each semi-structured interview was guided by six main interview questions, as illustrated in the interview map below (please see Figure 1).

### Data analysis

The SPSS software was utilized to analyze the quantitative data collected, encompassing mindfulness, acceptance, performance-related satisfaction, and athletes’ training performance. Data analysis was conducted using a 2×3 mixed-design ANOVA, as per the experimental design.

Audio recordings were made of all interviews, followed by verbatim transcription of the interview records. A combination of inductive and deductive thematic analysis, commonly utilized for qualitative coding and analysis (Braun and Clarke, 2006), was employed to analyze the collected qualitative data, namely the interview transcripts. Inductive thematic analysis was particularly apt for uncovering athletes’ experiences with the MAIC training, allowing for the generation of cohesive and descriptive themes that closely reflected their detailed experiences and perceptions (Braun and Clarke, 2006). This approach, combined with subsequent deductive thematic analysis, facilitated the integration of conceptual propositions regarding athletes’ receptiveness and perceptions of the MAIC training (Fereday and Muir-Cochrane, 2006). For details of this approach, the initial inductive thematic analysis identified key themes emerging from the athletes’ narratives. To further refine and interpret these themes within the theoretical framework of mindfulness and acceptance, a deductive approach was employed. Coding categories were developed based on these three needs and applied them to the existing themes, examining how the athletes’ experiences reflected their sense of autonomy in choosing to engage with the MAIC program, their perceived competence in applying mindfulness and acceptance skills, and their feelings of connection and belonging within the training environment. This deductive analysis resulted in a more nuanced understanding of the athletes’ receptiveness and perceptions of the MAIC training, highlighting the role of psychological need satisfaction in their engagement and perceived benefits of the program. The iterative nature of this process involved the emergence of meaning codes from each transcript, with themes evolving through comparisons of these codes. These themes were rigorously discussed with a distinguished Chinese expert in sport applied psychology and mindfulness and acceptance-based training and two coaches of the athletes participating in the MAIC training, with iterations as needed. Through the iterative process of inductive and deductive thematic analysis, a hierarchical structure consisting of lower order themes, higher-order themes, and general dimensions was identified. The lower order themes represented specific aspects of the athletes’ experiences, such as their initial curiosity about mindfulness, challenges in applying acceptance during competitions, and perceived improvements in focus and emotional regulation. These lower order themes were then grouped into higher-order themes based on shared concepts and relationships. Finally, the higher-order themes were organized into general dimensions reflecting the athletes’ overall receptiveness and perceptions of the MAIC training.

## Results of quantitative analysis

The SPSS 25 statistical software package was used to conduct the data analyses for the RCT. Preliminary analyses were conducted to screen the collected data, revealing no missing data and no significant differences (*p* > 0.05) across all variables between both groups prior to the MAIC training. A 2 (group) x 3 (data point) mixed-design ANOVA (i.e., two-way repeated measures analyses of variance) was performed to assess the impact of experimental conditions (i.e., between-subjects independent variable with 2 levels: MT group and CG group) and data points (i.e., within-subjects independent variable with 3 levels: pre-, post-, and follow-up of the MAIC training) on athletes’ mindfulness, acceptance, performance-related satisfaction, coach-rating training performance, and self-rating training performance. Subsequently, in further *post hoc* analyses, one-way repeated measures ANOVA was conducted to explore within-subjects differences between the three data points for both groups. Descriptive statistics (including mean, standard deviation, and Cronbach’s *α*) of the targeted variables for both groups at the three data points were presented in Table 2.

**Table 2:** Descriptive statistics of the aimed variables at three data points.

Variables	Pre-			Post-			Follow-up		
	M	SD	α	M	SD	α	M	SD	α
Mindfulness									
MT	53.85	7.05	0.94	62.80	6.78	0.87	61.80	7.02	0.92
CG	53.70	7.22	0.80	53.70	6.91	0.82	53.65	7.43	0.86
Acceptance									
MT	22.55	7.53	0.91	15.70	6.16	0.93	19.50	6.13	0.92
CG	22.50	7.41	0.83	22.60	7.42	0.87	22.55	7.04	0.86
Satisfaction									
MT	25.95	3.78	0.81	33.35	3.87	0.82	30.50	3.72	0.80
CG	25.90	3.74	0.79	26.10	3.81	0.77	26.00	3.83	0.76
CR performance									
MT	21.20	2.93		25.40	1.90		23.10	2.71	
CG	21.35	3.01		21.20	2.84		21.40	2.91	
SR performance									
MT	19.80	3.00		24.15	2.72		22.20	2.65	
CG	19.85	3.10		20.05	2.98		20.00	3.04	

*M*, Mean; SD, standard deviation; *α*, Cronbach’s Alpha; CR performance, coachrating performance; SR performance, self-rating performance.

### Mindfulness

The results of the 2 × 3 mixed-design ANOVA for mindfulness scores unveiled a significant interaction effect (group x data point) with *F*_(2, 76)_ = 275.79, *p* < 0.001, Partial *η^2^* = 0.88. Moreover, the main effect of groups was significant [*F*_(1, 38)_ = 6.81, *p* = 0.013, Partial *η^2^* = 0.15], indicating a notable between-group difference. Similarly, the main effect of data points was significant [*F*_(2, 76)_ = 273.14, *p* < 0.001, Partial *η^2^* = 0.88], highlighting a significant within-group difference.

*Post hoc* independent-samples *t*-tests (Table 3) revealed significantly higher mindfulness scores for the MT group compared to the CG group at both post- and follow-up data points, indicating a consistently elevated mindfulness level in the MT group.

**Table 3:** Summaries of *post hoc* independent-samples *t*-test.

Variables	Pre			Post				Follow-up			
	t	p	df	t	p	df	95%CI	t	p	df	95%CI
Mindfulness	0.07	0.947	38	4.20	<0.001	38	[4.72, 13.48]	3.56	0.001	38	[3.52, 12.78]
Acceptance	0.02	0.983	38	−3.20	0.003	38	[−11.26,-2.54]	−1.46	0.152	38	[−7.28, 1.18]
Satisfaction	0.04	0.967	38	5.97	<0.001	38	[4.79, 9.71]	3.77	0.001	38	[2.08, 6.92]
CR performance	−0.16	0.874	38	5.50	<0.001	38	[2.65, 5.75]	1.91	0.064	38	[−0.10, 3.50]
SR performance	−0.05	0.959	38	4.54	<0.001	38	[2.27, 5.93]	2.44	0.020	38	[0.37, 4.03]

*t*, *t* value; *p*, *p* value; df, degree of freedom; CI, confidence interval; CR performance, coach-rating performance; SR performance, self-rating performance.

Further *post hoc* one-way repeated measures ANOVA for the MT group demonstrated a significant difference across the three data points [*F*_(2, 38)_ = 686.31, *p* < 0.001, Partial *η^2^* = 0.97]. Pairwise comparisons (Table 4) indicated significantly higher mindfulness scores at post- and follow-up compared to pre-, with post- scores also significantly higher than follow-up scores. These findings suggest a significant improvement in mindfulness levels for the MT group post- and follow-up compared to pre-, with post- showing superior improvement over follow-up.

**Table 4:** Summaries of *post hoc* pairwise comparisons.

Variables	Pre- vs. Post-		Pre- vs. Follow-up		Post- vs. Follow-up	
	M diff	p	M diff	p	M diff	p
MT						
Mindfulness	−8.95	<0.001	−7.95	<0.001	1.00	<0.001
Acceptance	6.85	<0.001	3.05	<0.001	−3.80	<0.001
Satisfaction	−7.40	<0.001	−4.55	<0.001	2.85	<0.001
CR performance	−4.20	<0.001	−1.90	<0.001	2.30	0.001
SR performance	−4.35	<0.001	−2.40	<0.001	1.95	<0.001
CG						
Mindfulness	0.00	1.00	0.05	0.84	0.05	0.88
Acceptance	−1.00	0.541	−0.05	0.772	0.05	0.804
Satisfaction	−0.20	0.214	−0.10	0.33	0.10	0.494
CR performance	0.15	0.267	−0.05	0.748	−0.20	0.258
SR performance	−0.20	0.258	−0.15	0.379	0.05	0.789

*M diff*, mean difference; *p*, *p* value; CR performance, coach-rating performance; SR performance, self-rating performance.

Conversely, for the CG group, *post hoc* one-way repeated measures ANOVA revealed no significant difference across the three data points [*F*_(2, 38)_ = 0.016, *p* = 0.98, Partial *η^2^* = 0.001]. Pairwise comparisons (refer to Table 4) also indicated no significant differences in mindfulness scores between any of the three data points for the CG group, indicating a consistent mindfulness level across all data points.

### Acceptance

The 2 × 3 mixed-design ANOVA for the scores of the CV-AAQ II (experiential avoidance scores) unveiled a significant interaction effect (group x data point) between groups and data points, with *F*_(2, 76)_ = 38.58, *p* < 0.001, Partial *η^2^* = 0.50. Furthermore, the main effect of data points was significant [*F*_(2, 76)_ = 36.40, *p* < 0.001, Partial *η^2^* = 0.49], indicating a significant within-group difference. However, the main effect of groups was not significant [*F*_(1, 38)_ = 2.34, *p* = 0.134, Partial *η^2^* = 0.058].

*Post hoc* independent-samples *t*-tests (Table 3) revealed that experiential avoidance scores of the MT group were significantly lower than the CG group only at the post-data point, without follow-up. This suggests that the acceptance level of the MT group was significantly higher than the CG group only immediately after the intervention.

*Post hoc* one-way repeated measures ANOVA for the MT group demonstrated a significant difference across the three data points [*F*_(2, 38)_ = 39.45, *p* < 0.001, Partial *η^2^* = 0.68]. Pairwise comparisons (refer to Table 4) indicated significantly lower experiential avoidance scores at post- and follow-up compared to pre-, with post- scores also significantly lower than follow-up scores. This suggests that the acceptance level of the MT group at both post- and follow-up was significantly higher than pre-, with post- showing superior improvement over follow-up.

In contrast, for the CG group, *post hoc* one-way repeated measures ANOVA revealed no significant difference across the three data points [*F*_(2, 38)_ = 0.16, *p* = 0.853, Partial *η^2^* = 0.008]. Pairwise comparisons (refer to Table 4) also indicated no significant differences in experiential avoidance scores between any of the three data points for the CG group, indicating a consistent acceptance level across all data points.

### Satisfaction

The 2 × 3 mixed-design ANOVA analysis of performance-related satisfaction scores unveiled a significant interaction effect (group x data point) between groups and data points, with *F*_(2, 76)_ = 667.36, *p* < 0.001, Partial *η^2^* = 0.95. Furthermore, both the main effects of groups and data points were significant, with *F*_(1, 38)_ = 10.87, *p* = 0.002, Partial *η^2^* = 0.22, and *F*_(2, 76)_ = 742.18, *p* < 0.001, Partial *η^2^* = 0.95, respectively. These results indicate a significant between-group difference and a significant within-group difference, respectively.

*Post hoc* independent-samples t-tests (refer to Table 3) revealed significantly higher satisfaction scores for the MT group compared to the CG group at both post- and follow-up data points, suggesting a consistently elevated satisfaction level in the MT group.

Further analysis via *post hoc* one-way repeated measures ANOVA for the MT group demonstrated a significant difference across the three data points [*F*_(2, 38)_ = 1307.09, *p* < 0.001, Partial *η^2^* = 0.99]. Pairwise comparisons (refer to Table 4) indicated significantly higher satisfaction scores at post- and follow-up compared to pre-, with post- scores also significantly higher than follow-up scores. This suggests that the satisfaction level of the MT group at both post- and follow-up was significantly higher compared to pre-, with post- showing superior improvement over follow-up.

Conversely, *post hoc* one-way repeated measures ANOVA for the CG group revealed no significant difference across the three data points [*F*_(2, 38)_ = 1.10, *p* = 0.344, Partial *η^2^* = 0.055]. Pairwise comparisons (refer to Table 4) also indicated no significant differences in satisfaction scores between any of the three data points for the CG group, indicating a consistent satisfaction level across all data points.

### Coach-rating performance

The results of the 2 × 3 mixed-design ANOVA for coach-rating sport training performance scores unveiled a significant interaction effect (group x data point) between groups and data points, with *F*_(2, 76)_ = 26.13, *p* < 0.001, Partial *η^2^* = 0.41. Additionally, the main effect of groups was significant [*F*_(1, 38)_ = 5.82, *p* = 0.021, Partial *η^2^* = 0.13], indicating a notable between-group difference. Similarly, the main effect of data points was significant [*F*_(2, 76)_ = 22.49, *p* < 0.001, Partial *η^2^* = 0.37], highlighting a significant within-group difference.

*Post hoc* independent-samples t-tests (refer to Table 3) revealed significantly higher coach-rating performance scores for the MT group compared to the CG group at both post- and follow-up data points, indicating superior coach-rating performance in the MT group.

Further analysis via *post hoc* one-way repeated measures ANOVA for the MT group demonstrated a significant difference across the three data points [*F*_(2, 38)_ = 25.91, *p* < 0.001, Partial *η^2^* = 0.58]. Pairwise comparisons (refer to Table 4) indicated significantly higher coach-rating performance scores at post- and follow-up compared to pre-, with post- scores also significantly higher than follow-up scores. This suggests that the coach-rating performance of the MT group at both post- and follow-up was significantly better compared to pre-, with post- showing superior improvement over follow-up.

Conversely, *post hoc* one-way repeated measures ANOVA for the CG group revealed no significant difference across the three data points [*F*_(2, 38)_ = 0.93, *p* = 0.405, Partial *η^2^* = 0.046]. Pairwise comparisons (refer to Table 4) also indicated no significant differences in coach-rating performance between any of the three data points for the CG group, indicating a consistent coach-rating performance level across all data points.

### Self-rating performance

The 2 × 3 mixed-design ANOVA analysis for self-rating sport training performance scores demonstrated a significant interaction effect (group x data point) between groups and data points, with *F*_(2, 76)_ = 151.39, *p* < 0.001, Partial *η^2^* = 0.80. Furthermore, the main effect of groups was significant [*F*_(1, 38)_ = 5.20, *p* = 0.028, Partial *η^2^* = 0.12], indicating a significant between-group difference. Similarly, the main effect of data points was significant [*F*_(2, 76)_ = 182.43, *p* < 0.001, Partial *η^2^* = 0.83], highlighting a significant within-group difference.

*Post hoc* independent-samples t-test analysis results (refer to Table 3) revealed significantly higher self-rating performance scores for the MT group compared to the CG group at both post- and follow-up data points, indicating superior self-rating performance in the MT group.

Further analysis via *post hoc* one-way repeated measures ANOVA for the MT group demonstrated a significant difference across the three data points [*F*_(2, 38)_ = 357.24, *p* < 0.001, Partial *η^2^* = 0.95]. Pairwise comparisons (refer to Table 4) indicated significantly higher self-rating performance scores at post- and follow-up compared to pre-, with post- scores also significantly higher than follow-up scores. This suggests that the self-rating performance of the MT group at both post- and follow-up was significantly better compared to pre-, with post- showing superior improvement over follow-up.

Conversely, *post hoc* one-way repeated measures ANOVA analysis for the CG group revealed no significant difference across the three data points [*F*_(2, 38)_ = 0.71, *p* = 0.497, Partial *η^2^* = 0.04]. Pairwise comparisons (refer to Table 4) also indicated no significant differences in self-rating performance between any of the three data points for the CG group, suggesting a consistent self-rating performance level across all data points.

## Results of qualitative exploration

All audio recordings of the 14 athletes from the MT group, who volunteered for interviews, were transcribed verbatim, resulting in 266 A4 pages of single-spaced transcripts. Thematic analysis of these transcripts revealed four general dimensions pertaining to the athletes’ experiences (i.e., receptiveness and perceptions) of the MAIC training, which include: (a) Attitude towards the MAIC training, (b) Reflection on the MAIC learning process, (c) Outcome of the MAIC training, and (d) Recommendation for future MAIC training. Within these dimensions, 11 higher-order themes emerged, comprised of 43 lower-order themes. Tables 5–8 present the four general dimensions along with the higher-order and lower-order themes contained within them, respectively. Additionally, the results include select quotes from the athletes to further illustrate the meaning of the themes.

**Table 5:** General dimension a: attitude towards the MAIC training (*n* = 14).

Higher order themes	Lower order themes
Appealing to learner (*n* = 14)	Big interest at beginning (*n* = 14)
	Continuously high interest during the process (*n* = 13)
	Further interest after the end (*n* = 11)

Beneficial training (*n* = 14)	Help with sport (*n* = 14)
	Help with daily life (*n* = 8)

Novel approach (*n* = 14)	Different from other approaches learnt before (*n* = 14)
	Fresh conception (*n* = 14)
	Fresh skills (*n* = 12)
	Fresh mechanisms (*n* = 11)

Willing to apply (*n* = 14)	Use in sport (*n* = 14)
	Use in daily life (*n* = 10)
	Introduce to others to know and use (*n* = 9)

The higher order themes were demonstrated in alphabetical order; *n* refers to the number of the athletes that mentioned the themes in the interview.

**Table 6:** General dimension b: Reflection on the MAIC learning process (*n* = 14).

Higher order themes	Lower order themes
Challenge (*n* = 14)	Hectic schedule (*n* = 14)
	Environment (*n* = 12)
	Conception understanding (*n* = 12)
	Uncomfortable practice (*n* = 13)
Favorite (*n* = 14)	Clear framework and rundown (*n* = 14)
	Sessions implemented prior to daily training (*n* = 12)
	Pre- and post-session practice (*n* = 14)
	Coaches’ participation (*n* = 13)
	Q & A (*n* = 12)
	Breathing exercise (*n* = 14)

The higher order themes were demonstrated in alphabetical order; *n* refers to the number of the athletes that mentioned the themes in the interview.

**Table 7:** General dimension c: Outcome of the MAIC training (*n* = 14).

Higher order themes	Lower order themes
Acquired competence (*n* = 14)	Attentional focus (*n* = 14)
	Let go of internal experience (*n* = 9)
	Flexible thinking (*n* = 8)
	Mindful breathing (*n* = 14)
Impact on sport training and competition (*n* = 14)	Enhanced performance (*n* = 14)
	Increased concentration (*n* = 14)
	Increased feeling of poise (*n* = 14)
	Increased confidence (*n* = 10)
	Increased feeling of mastery (*n* = 9)
Influence on daily life (*n* = 10)	Ameliorate interpersonal relationship (*n* = 10)
	Optimize action efficacy (*n* = 8)
	Promote academic study (*n* = 7)

The higher order themes were demonstrated in alphabetical order; *n* refers to the number of the athletes that mentioned the themes in the interview.

**Table 8:** General dimension d: Recommendation for future MAIC training (*n* = 14).

Higher order themes	Lower order themes
Delivery of the MAIC (*n* = 14)	Smaller group (*n* = 11)
	Individual meeting (*n* = 9)
	Sport-specific cases (*n* = 12)
	More monitoring and feedback (*n* = 10)
	Longer program duration (*n* = 7)
Instruction after the end (*n* = 12)	Practice guide (*n* = 11)
	Application guide (*n* = 12)
	Evaluation guide (*n* = 9)
	Continuous communication with instructor (*n* = 12)

The higher order themes were demonstrated in alphabetical order; *n* refers to the number of the athletes that mentioned the themes in the interview.

### General dimension a: attitude towards the MAIC training

The general dimension (a) comprising four higher-order themes (refer to Table 5), namely (a) appealing to learner, (b) beneficial training, (c) novel approach, and (d) willingness to apply, encapsulates the attitudes of the interviewed athletes towards the MAIC training from their initial encounter to its conclusion.

#### Appealing to learner

As participants in the MAIC training, all 14 athletes who volunteered for interviews expressed strong interest from their initial exposure to the program during the first session. For example, one athlete remarked, “I distinctly recall during the first session, you dedicated significant time to introducing the content and potential benefits of the training. This introduction really sparked my interest.” (A1, where An refers to Athlete1 through Athlete14, according to the interview sequence.)

#### Beneficial training

After completing the MAIC training, all 14 interviewed athletes reported finding the training beneficial. In terms of their athletic pursuits, they unanimously expressed that the MAIC training positively impacted their training and competitive performance in sports. “It’s evident that my approach to training and competing shifted after learning this method, and it’s been as helpful as you described,” stated A2.

#### Novel approach

Being introduced to mindfulness and acceptance-based training for the first time, all 14 athletes interviewed perceived the MAIC training as a novel approach. They unanimously acknowledged that the MAIC training differed significantly from other approaches they had previously encountered. “Despite having some experience with mental training before, this time around felt completely fresh and distinct,” remarked A9.

#### Willingness to apply

During the interviews, all 14 athletes expressed their eagerness to apply what they had learned from the MAIC training in practice. As a mindfulness and acceptance-based program tailored for athletes, each of the 14 athletes affirmed their intention to incorporate MAIC techniques into their sport training and competitions. “Absolutely, I’m keen to use it. Particularly during our learning sessions, I felt a strong urge to implement each new technique I learned in both training and competition,” remarked A5.

### General dimension b: reflection on the MAIC learning process

General dimension b encapsulated the reflections of all 14 interviewed athletes on the learning process of the MAIC training, comprising two higher-order themes: (a) challenge and (b) favorite (please refer to Table 6).

#### Challenge

All 14 interviewed athletes noted encountering challenges during the MAIC training. The majority of these elite adolescent athletes were part-time athletes balancing their athletic commitments with full-time schooling. Even among those who were full-time athletes, some still had academic obligations through schools collaborating with the HKSI. Consequently, all 14 athletes reported that managing their hectic daily schedules posed a challenge to fully engage in the MAIC training.

#### Favorite

In addition to the challenges mentioned earlier, all 14 interviewed athletes also highlighted their favorite aspects during the MAIC training. Among these favored factors, the clear structure and outline of the MAIC training were consistently praised by all 14 athletes as conducive to their learning experience. For instance, one athlete remarked, “The training’s framework was very clear to me, particularly during the initial session. It provided a comprehensive overview of the training’s structure and its significance. I found that extremely beneficial.” (A1).

### General dimension c: outcome of the MAIC training

The third general dimension, the outcome of the MAIC training, encapsulated the main gains and impacts reported by all 14 interviewed athletes upon completing the training. This dimension comprised three primary higher-order themes: (a) Acquired competence, (b) Impact on sport training and competition, and (c) Influence on daily life (please refer to Table 7).

#### Acquired competence

By assimilating the knowledge and exercises provided in the MAIC training, the athletes developed specific competencies to address challenges both within and outside of sports. All 14 interviewed athletes reported acquiring competencies through the completion of the MAIC training. Among these competencies, maintaining attentional focus on current tasks and actions was highlighted by all 14 athletes. For instance, one athlete remarked, “Over time, I noticed an improvement in my ability to concentrate on the task at hand and remain focused on present actions.” (A3).

#### Impact on sport training and competition

In addition to the acquired competencies from the MAIC training, the impact on athletes’ sport training and competition became evident. All 14 interviewed athletes reported experiencing some degree of impact on their sport training and competition as a result of completing the MAIC training. The most notable effect was the enhancement of athletes’ performance in both training sessions and competitions, a sentiment echoed by all 14 athletes. For instance, one athlete remarked, “I’ve noticed myself becoming more engaged in my training, and the effectiveness of my workouts has improved compared to before.” (A2).

#### Influence on daily life

In addition to the impacts on athletes’ sport training and competition, a portion of the interviewed athletes—ten out of fourteen—reported that the MAIC training also significantly influenced their daily lives, particularly in terms of interpersonal relationships, actions in normal life, and academic study. By completing the MAIC training, athletes found themselves becoming more patient and compassionate, which in turn improved their interpersonal relationships with others, particularly with close individuals such as parents and friends. “During interactions with my friends, I can sense that I’ve become kinder and more pleasant, not only to them but also to myself,” stated A1.

### General dimension d: recommendation for future MAIC training

General dimension d, comprising two higher-order themes (please refer to Table 8), encapsulated the advice offered by the interviewed athletes for enhancing future MAIC training, focusing on the delivery of the training and necessary guidance following its conclusion.

#### Delivery of the MAIC

To enhance the effectiveness of future MAIC training, all 14 interviewed athletes provided their suggestions on the format and delivery of the training. A significant number of athletes—eleven out of fourteen—expressed a preference for smaller group sessions. One athlete stated, “I’d prefer the sessions to be conducted in smaller groups.” (A13).

#### Instruction after the end

In addition to suggestions regarding training delivery in the future, the majority of athletes interviewed—twelve out of fourteen—also recommended that the instructor provide necessary instructions related to practice, application, evaluation, and communication after the conclusion of the MAIC training. Despite completing the training sessions, the athletes believed they still required guidance on sustaining mindful practices independently, applying what they had learned in real-life scenarios, and periodically evaluating their progress. Eleven out of fourteen athletes expressed a desire for self-guided practice instructions to ensure they could continue practicing and maintain effectiveness after the sessions ended. “After completing the training, I believe it’s important to have some guidelines for self-practice to ensure we can continue practicing and maintain effectiveness,” noted A10.

## Discussion

### The effectiveness of the MAIC training program

The aim of this study was to employ a mixed-method approach, incorporating both quantitative and qualitative methods, to further evaluate the effectiveness of MIAC (Mindfulness, Acceptance, and Internal Commitment) training and to gain a comprehensive understanding of the experiences of elite adolescent athletes from Hong Kong who participated in this training. The quantitative aspect, utilizing a Randomized Controlled Trial (RCT), revealed significant enhancements in mindfulness, acceptance, performance-related satisfaction, and sports training performance among participants in the MIAC training (MT) group compared to the control group. Moreover, within the MT group, these factors showed significant improvement both immediately after the training and at the follow-up assessment compared to pre-training levels. In the qualitative phase, semi-structured interviews were conducted with 14 athletes from the MT group who volunteered, revealing four main themes: attitudes towards the MIAC training, reflections on the learning process, outcomes gained from the training, and recommendations for future iterations of the program. These qualitative insights complemented the quantitative findings, consistently highlighting the effectiveness of MIAC training and the positive reception and perceptions of the athletes involved. These combined results contribute valuable evidence supporting the efficacy of MIAC training in enhancing Chinese athletes’ performance and their internal experiences, such as thoughts and emotions. Additionally, they lend further support to previous research in sport psychology regarding mindfulness and acceptance-based interventions [e.g., Birrer et al. (2012), Gardner and Moore (2012), and Lindsay and Creswell (2017)]. Moreover, the study offers substantial insights and suggestions that can inform the refinement and application of MIAC training programs moving forward.

### Persistence of regular mindfulness practice and different types of mindfulness practice

One of the primary objectives of the Mindfulness, Acceptance, and Internal Commitment (MAIC) training, tailored specifically for Chinese athletes, is to foster mindfulness among participants (Si et al., 2014; Su et al., 2019). As anticipated, the findings of Study II revealed improvements in athletes’ mindfulness levels following participation in the MAIC training, consistent with previous research on the topic [e.g., Bu and Si (2014), Bu (2015), Liu et al. (2016), Si et al. (2016), and Zhang et al. (2016)]. However, this study also identified certain challenges in cultivating athletes’ mindfulness that merit further discussion. In the present study, while the mindfulness levels of athletes in the MAIC training (MT) group significantly surpassed those of the control group post-training, they experienced a notable decline at the follow-up assessment compared to the immediate post-training measurement. A similar phenomenon was observed in a prior study (Zhang et al., 2016) on MAIC training, with poor adherence to home-based mindfulness practice cited as a primary explanation (Rosenzweig et al., 2010). This study echoes this observation and underscores the importance of emphasizing the sustained practice of mindfulness.

In addition to poor adherence, the decline in mindfulness may also be influenced by the types of mindfulness practices and the characteristics of adolescent athletes from Hong Kong. Currently, three main types of mindfulness practices are extensively researched: focused attention (FA), open monitoring (OM), and loving-kindness (LK) meditation (Lippelt et al., 2014). FA involves maintaining focused attention on a chosen object or event, such as the breath, to prevent the mind from wandering (Tops et al., 2014). Conversely, OM requires practitioners to remain in a monitoring state, attentively observing any experiences without selection or judgment (Lippelt et al., 2014). While FA is generally more accessible for novice practitioners, OM demands a higher level of engagement. Based on our qualitative observations, which align with the characteristics of adolescent athletes from Hong Kong, we noted that participants appeared to favor FA-related practices such as mindful breathing exercises, which may be perceived as more accessible and easier to grasp. However, they struggled with OM-related practices, such as body scan exercises, which demanded more effort. Furthermore, factors like limited living space in Hong Kong and the hectic schedules of adolescent athletes may have contributed to poor adherence to regular practice after the training period. Moreover, the eight-week duration of the MAIC training program in this study might not have provided adequate time for adolescent athletes to sufficiently advance to OM practices. This discrepancy was reflected in athletes’ recommendations during interviews to extend the duration of the training. Collectively, these factors likely contributed to the decline in athletes’ mindfulness following the conclusion of the MAIC training program.

### Cultivating acceptance: long time work

Acceptance, as another fundamental element of existing mindfulness training interventions (Chambers et al., 2009), is intricately linked with and complements mindfulness (Lindsay and Creswell, 2017). The enhancement of mindfulness can facilitate experiential acceptance (Gardner and Moore, 2007; Zhang et al., 2016), a finding corroborated by the results of the present study. Here, the level of acceptance among athletes engaged in the Mindfulness, Acceptance, and Internal Commitment (MAIC) training significantly improved immediately after the training compared to the control group. However, no significant difference was observed between the MAIC training (MT) group and the control group at the follow-up assessment. In a prior study focusing on MAIC training among free combat athletes from Mainland China (Bu and Si, 2014), the improvement in acceptance was not as pronounced as anticipated. This discrepancy was attributed to two primary reasons: firstly, acceptance was introduced relatively late in the training, allowing insufficient time for participants to develop it adequately; secondly, acceptance proved challenging for participants to grasp. Unlike some mindfulness skills, such as focused attention (FA), which may exhibit immediate improvements post-practice, acceptance often requires more time to cultivate (Lindsay and Creswell, 2017). Moreover, certain mindfulness skills, like open monitoring (OM), serve as foundational elements for cultivating acceptance. Hence, mindfulness is typically introduced prior to acceptance in mindfulness and acceptance-based training programs. Furthermore, acceptance, being a broad construct encompassing various dimensions (e.g., nonjudgment, nonreactivity, openness) distinct from traditional psychological skills training (PST), may appear abstract and ambiguous to athletes, necessitating additional effort and time to comprehend (Josefsson et al., 2019). Considering these factors, it becomes apparent why athletes’ levels of acceptance did not improve as expected following the conclusion of the MAIC training in the current study. Similar to mindfulness, acceptance requires dedicated time and practice for athletes to understand and apply effectively, especially for adolescent athletes whose personal characteristics, such as perseverance and logical thinking, are still maturing.

### Potential explanation on the improvement of the performance

The cultivation of a mindful mindset, coupled with improvements in mindfulness and acceptance, can create favorable conditions for optimal sports performance (Josefsson et al., 2019). Numerous studies have demonstrated enhancements in sport performance, as assessed by subjective measures such as coach ratings or self-ratings, following participation in mindfulness and acceptance-based training (e.g., Gardner and Moore, 2007; Lutkenhouse, 2007). Previous research on the Mindfulness, Acceptance, and Internal Commitment (MAIC) training has also indicated improvements in sport performance among participants (Bu and Si, 2014; Bu, 2015; Liu et al., 2016; Si et al., 2016; Zhang et al., 2016). Consistent with these findings, the current study observed significant improvements in athletes’ training performance following MAIC training. Furthermore, as training performance improved, athletes reported increased satisfaction with their performance. Although the underlying psychological mechanisms linking mindfulness to performance outcomes require further exploration (Lutz et al., 2015; Lindsay and Creswell, 2017), certain components of mindfulness, such as focused attention (FA) and acceptance, have been associated with behavioral enhancements (Chambers et al., 2009). Focusing attention on the present task may help athletes mitigate distractions that could hinder performance (Gardner and Moore, 2012). Additionally, mindful acceptance, which involves changing athletes’ relationship to internal experiences (e.g., thoughts, emotions, and feelings), may enable more efficient use of mental resources during training or competition (Moore, 2009; Gardner and Moore, 2012). Moreover, according to athletes’ feedback in interviews, improved abilities in flexible thinking and letting go of internal experiences may enhance concentration, confidence, and composure, facilitating optimal decision-making in challenging situations during training or competition (Josefsson et al., 2019). Furthermore, in the current study, all conceptual education and practical sessions were conducted prior to athletes’ daily sport training sessions, potentially serving as a significant facilitator for improved performance and satisfaction. However, following the conclusion of MAIC training, declines in mindfulness and acceptance levels corresponded with setbacks in both athletes’ training performance and performance-related satisfaction at the follow-up assessment. To effectively assimilate and apply what they have learned from mindfulness and acceptance-based training, athletes, particularly adolescents, may require an extended period of practice and evaluation to sustain or strengthen the effects on their performance (Bühlmayer et al., 2017; Josefsson et al., 2019).

### Effectiveness of the MAIC training program outside of sport

Mindfulness and acceptance-based interventions not only yield benefits in sports but also offer valuable applications beyond athletic endeavors (Cote et al., 2019). Existing qualitative studies on these interventions have demonstrated their positive impact on participants’ academic pursuits, personal development, interpersonal relationships, work-life balance, overall well-being, mental health, and life satisfaction outside of sporting contexts (Longshore and Sachs, 2015; Cote et al., 2019). Furthermore, a randomized controlled trial (RCT) study on the Mindfulness, Acceptance, and Internal Commitment (MAIC) training provided evidence of its effectiveness in enhancing the motor skills learning of beginners (Zhang et al., 2016). The qualitative findings of the current study further support the notion that MAIC training can benefit athletes beyond the realm of sports. Improved levels of mindfulness and acceptance, coupled with the acquisition of specific skills such as flexible thinking and letting go of internal experiences, were reported to enhance academic performance, action efficacy, and interpersonal relationships in athletes’ daily lives. Given the effectiveness of mindfulness and acceptance-based interventions in domains outside of sports, MAIC training tailored for Chinese athletes and applied in sports training and competition may be transferable to athletes’ daily lives. This is particularly beneficial for adolescent athletes who often juggle demanding schedules encompassing both sports and academic studies. By applying the principles learned from MAIC training, these athletes can achieve balance between their athletic pursuits and daily life responsibilities, facilitating effective and efficient actions both on and off the field.

## Conclusion and implication

Since the inception of the Mindfulness, Acceptance, and Internal Commitment (MAIC) training program tailored for Chinese athletes in 2014, six years have elapsed. Over this period, a series of applied studies involving elite athletes from various sports and regions of China have investigated and substantiated the effectiveness of the MAIC training [e.g., Bu and Si (2014), Bu (2015), Liu et al. (2016), Si et al. (2016), Zhang et al. (2016), and ]. Consistent with these prior investigations, the present research provides further support for the efficacy of MAIC training in enhancing relevant psychological factors, such as mindfulness, acceptance, and performance-related satisfaction, as well as improving sport performance, specifically among adolescent elite athletes from Hong Kong. Furthermore, through qualitative exploration of athletes’ experiences with the MAIC training, the current study gained a nuanced understanding of their genuine receptiveness and perceptions towards the program. The qualitative findings align with and reinforce the quantitative outcomes of this research while also shedding light on areas for improvement within the MAIC training program and offering tangible suggestions for its further development. Despite obtaining preliminary evidence supporting the effectiveness of MAIC training for Chinese elite athletes, there remain limitations inherent to the training and research on it that require attention in future endeavors. Just as cultivating athletes’ mindfulness cannot be achieved in a single action, the MAIC training, as a mindfulness and acceptance-based program tailored for Chinese athletes, necessitates ongoing refinement and development (Si et al., 2020).

## Limitation and future direction

### Limitation

While this study provides valuable insights into the efficacy of the MAIC training program for adolescent elite athletes, it is essential to acknowledge certain limitations:

#### Sample size

The relatively small sample size, while typical for studies involving elite athletes, limits the generalizability of the findings to broader populations. Future research with larger and more diverse samples is needed to confirm the program’s effectiveness across different athlete groups.

#### Follow-up duration

The observed diminishing effects at the two-month follow-up raise questions about the long-term sustainability of the intervention’s benefits. Extending the follow-up period in future studies would provide valuable data on the program’s lasting impact.

#### Control group

The absence of an active control group makes it difficult to definitively isolate the specific effects of MAIC training from other potential influences. Implementing an active control group engaging in an alternative intervention could strengthen causal inferences in future studies.

#### Qualitative participation

While valuable insights were gained, the voluntary nature of the interview process and the resulting smaller sample size within the qualitative component may introduce some bias. Exploring strategies to increase participation in future qualitative studies would enhance the richness and representativeness of the data.

### Future direction

Building upon the current study’s findings, several avenues for future research can further enhance our understanding and application of MAIC training:

#### Long-term effects

Investigating the sustained impact of MAIC training on athletes’ psychological well-being and performance over extended periods, beyond the two-month follow-up employed in this study, is crucial to assess the program’s long-term efficacy.

#### Diverse populations

Exploring the program’s effectiveness across diverse athlete populations, encompassing various age groups, skill levels, and cultural backgrounds, will provide valuable insights into its generalizability and potential for adaptation to specific contexts.

#### Comparative effectiveness

Comparing MAIC training to other established interventions or active control conditions will help determine its unique contributions and comparative effectiveness in enhancing athletes’ psychological functioning and performance outcomes.

#### Underlying mechanisms

Examining the potential psychological mechanisms underlying the observed effects of MAIC training, such as changes in attentional processes, emotion regulation skills, or self-compassion, can provide a deeper understanding of how the program exerts its influence on athletes.

#### Qualitative exploration

Continued utilization of qualitative research methods can offer richer insights into athletes’ experiences with MAIC training, allowing for program tailoring and refinement to better address their specific needs and challenges.

## Data availability statement

The raw data supporting the conclusions of this article will be made available by the authors, without undue reservation.

## Ethics statement

The studies involving humans were approved by the ethics committee of the Education University of Hong Kong. The studies were conducted in accordance with the local legislation and institutional requirements. Written informed consent for participation in this study was provided by the participants’ legal guardians/next of kin.

## Author contributions

NS: Conceptualization, Data curation, Formal Analysis, Funding acquisition, Investigation, Methodology, Project administration, Resources, Supervision, Validation, Writing – original draft, Writing – review & editing. GS: Conceptualization, Investigation, Methodology, Project administration, Resources, Supervision, Validation, Writing – review & editing. WL: Data curation, Formal Analysis, Methodology, Validation, Writing – review & editing. DB: Data curation, Formal Analysis, Methodology, Writing – review & editing. XJ: Investigation, Resources, Supervision, Writing – review & editing.
